# Asylum Workers' Union

**Published:** 1917-07-21

**Authors:** 


### Asylum Workers' Union.

### MANIFESTO ON RECOGNITION.

The decision of the Lancashire Asylums Board not officially to recognise the National Asylum Workers' Union has been followed by the issue of a manifesto in the form of a long communication from the secretary of the Union, which includes the following passages:

"The [Lancashire Asylums] Board members themselves are members of employers' trade unions, and the medical officers of the asylums are all members of their trades union, and both parties claim the fullest recognition for their respective unions, yet the Board refuses the same elementary right to its subordinate asylums staffs. Sir Wm. Scott Barrett said: 'The committee felt sure that the staff would receive much more sympathetic treatment if they approached the committee in the old friendly way.' As a matter of fact, the asylums staffs are not permitted to approach their committees in a manner friendly or otherwise. . . . There was one occasion, so far as I can recollect, and that was when the Rainhill Asylum staff adopted the stay-in strike some little time ago. What the committees won't concede to reason they will concede to force; and I would advise them not to drive the lesson home too frequently.

"Under the present administrative system the asylums staffs in Lancashire must present all their grievances and applications to the committees through the medical superintendents. . . . It appears to be the case in Lancashire that the Asylums Board and its sub-committees are afraid to face facts, as these could be presented by their staffs or by their staffs' representatives. Instead, they shelter themselves behind the coat-tails of the medical superintendents, who consequently come in for a large amount of perhaps unmerited opprobrium, and this does not help to maintain friendly relations between these officials and the subordinate staffs.

"The plaintive plea that the concession of official recognition of the union would interfere with the heavy responsibility of the medical superintendents is obviously nonsensical, because, on the contrary, it would relieve them of a tremendous amount of responsibility by transferring to the visiting committees the duty which they ought never to have delegated to their subordinates, or to have neglected as they have hitherto done, of themselves dealing directly with the entire subject of wages and conditions of employment of their staffs. It is also untrue and absurd to suggest that official recognition of the union would create administrative difficulties; it would help to smooth over the obstacles. In the West Riding of Yorkshire asylums official recognition of the union was conceded to allay the alarming amount of unrest which prevailed for want of it. At those asylums to-day the staffs are the most contented in the country, because they are justly and openly dealt with through their union."

